# Breast tumor segmentation and morphological feature-based classification in ultrasound using a two-stage U-net and SVM

**DOI:** 10.3389/fbioe.2026.1774371

**Published:** 2026-03-06

**Authors:** Yang Ye, Mingtao Ye, Huihui Wang, Jiayu Fang, Guodao Zhang, Genfu Yang, Shurong Shen, Xiaoyang Li

**Affiliations:** 1 Department of Thyroid and Breast Surgery, Wenzhou Central Hospital, Affiliated to Wenzhou Medical University, Wenzhou, China; 2 School of Computer Science, Hangzhou Dianzi University, Hangzhou, China; 3 School of Chinese Medicine,Wenzhou Medical University, Wenzhou, China; 4 Institute of Intelligent Media Computing, Hangzhou Dianzi University, Hangzhou, China; 5 Key Laboratory of Micro-nano Sensing and IoT of Wenzhou, Wenzhou Institute of Hangzhou Dianzi University, Wenzhou, China; 6 Shangyu Institute of Science and Engineering Co.Ltd. Hangzhou Dianzi University, Shaoxing, China

**Keywords:** breast cancer, computer-aided diagnosis, morphological features, semantic segmentation, SVM, ultrasound imaging, U-net

## Abstract

**Introduction:**

Breast cancer remains one of the most prevalent and life-threatening conditions among women worldwide, making early detection and accurate diagnosis essential. In this study, we present a two-stage computer-aided diagnosis framework designed for the automated analysis of breast ultrasound images.

**Methods:**

The proposed system first employs a U-Net-based semantic segmentation model to detect and localize potential tumor regions. The model is trained and evaluated on a comprehensive dataset comprising normal, benign, and malignant cases. For each input image, the U-Net predicts a binary tumor mask; images with no detected tumor regions are classified as normal and excluded from further analysis. In the second stage, images identified as tumor-bearing undergo feature extraction to characterize the shape and morphology of the segmented tumor. Specifically, four handcrafted features—circularity, solidity, eccentricity, and extent—are computed from the predicted masks. These features are then used to train a support vector machine (SVM) classifier that distinguishes between benign and malignant tumors.

**Results:**

The segmentation model achieved an average Mask Intersection over Union% (Mask IoU) score of 91%, while the classification model reached an accuracy of 98.23% on the training set and 97.42% on the test set.

**Discussion:**

Unlike end-to-end deep learning approaches that often function as black boxes with limited clinical interpretability, our two-stage framework combines accurate deep learning-based segmentation with lightweight, handcrafted morphological feature classification using support vector machine. This design achieves high performance while preserving explainability through clinically meaningful shape descriptors, making it particularly suitable for real-world clinical deployment.

## 1 Introduction

Breast cancer is one of the most common cancer-induced causes of mortality in females across the globe. The early detection and accurate classification of lesions in the breast are vital in order to determine effective treatment and better patient outcomes. Although mammography is the most frequently utilized technique in cancer screening of the breast, ultrasound is increasingly gaining momentum due to its non-invasiveness, cost-effectiveness, and better performance on dense tissue. Ultrasonic evaluation is not subject to ionizing radiation and has the advantage of demonstrating masses not readily apparent by mammography. Nonetheless, the ultrasound image is highly operator dependent, subjective in nature, and subject to variability in distinguishing the benign and malignant lesions. These drawbacks emphasize the need for dependable, automated methods that aid clinicians in detection and diagnosis of the tumor. CAD systems have been introduced as effective tools in such situations, providing objective and reproducible decision support in medical imaging. Current CAD methods tend to be constrained by parameters like handcrafted feature dependency, non-generalizability, and low interpretability. The deep learning model, with CNNs, has been shown to possess great potential in addressing these drawbacks by learning relevant patterns from raw data. We present in this work a two-phase CAD system that incorporates deep learning-based segmentation with interpretable morphological analysis to effectively detect and classify the tumor in ultrasound images.

The evolution of deep learning methods has greatly improved the accuracy and efficiency of tumor classification and segmentation in medical imaging, especially in breast ultrasound (BUS) modalities. Current research has delved into different architectures and methodologies to enhance diagnosis performance. Zhou et al. (2021) presented a multi-task learning framework that jointly segments and classifies tumors from 3D automatic breast ultrasound images. They couple an encoder-decoder network and lightweight multi-scale network with feature refinement and iterative training. Chowdary et al. (2022) suggested an effective multi-task learning model for automatic segmentation and classification of ultrasound image-based breast tumors. They use an encoder-decoder architecture with bridge blocks for segmentation and dense branch for tumor classification with the objective of minimizing mortality due to early diagnosis. Islam et al. (2024) proposed ensemble deep convolutional neural network along with U-Net to improve segmentation and classification of mast carcinoma in ultrasound images. They show better performance in detection and classification of mammary cancer, demonstrating ensemble model superiority. Wei et al. (2024) introduced a new deep model termed multi-feature fusion multi-task (MFFMT) model for ultrasound image classification of breast tumor, with the focus on perception in the lesion region. They resolve challenges in extracting richer feature lesions and avoiding information-sharing conflicts to improve classification performance. Zhang et al. (2023) obtained fully automatic tumor segmentation of breast ultrasound images with deep learning. They attained state-of-the-art performance and demonstrated good transferability in external test sets, suggesting its application in auto-BUS health screening. Yang et al. (2023) suggested deep learning-based fast accurate segmentation and diagnosis of ultrasound images of mammary tumor by proposing two stages with semantic segmentation and classification of tumors. They use attention-based semantic segmentation followed by tumor classification and show good improvements in diagnosis speed and early screening rates. Lu et al. (2025) proposed multi-task learning network with object contextual attentions (MTL-OCA) with the use of simultaneous segmentation and classification in BUS images. They improve segmentation maps by learning pixel-region relationships with enhanced classification performance. Anari et al. (2025) proposed an interpretable attention-based model of breast tumor segmentation with the aid of the combination of UNet, ResNet, and DenseNet and EfficientNet. They utilized the best of both worlds in employing several frameworks in order to yield accurate and interpretable segmentations. Rastogi et al. (2025) addressed deep learning-integrated MRI brain tumor analysis including feature extraction, segmentation, and survival prediction utilizing replicator and volumetric networks. They highlighted the usability of deep learning in multimodal imaging settings. Yashaswini et al. (2025) demonstrated the usage of deep learning methods in the auto-segmentation of liver and liver tumor from CT images. With the help of a hybrid ResUNet model, their method tackles challenges in segmenting liver and tumor from CT images and proves the adaptability of deep learning across different types of image modalities.

Feature extraction from medical images is crucial in the diagnosis and prognosis of breast cancer. Quantifying morphology, textural, and radiomic characteristics provides deeper understanding of tumor behavior, supporting more precise assessments and patient-tailored treatment strategies. Xie et al. (2025) proposed a multi-dimensional radiomics framework to evaluate HER-2 status in breast cancer. Their technique demonstrated that the amalgamation of multiple image features improves diagnosis accuracy, with a non-invasive alternative to biopsy. Cai et al. (2025) used (Breast Imaging Reporting and Data System) BI-RADS characteristics from ultrasound images to distinguish between benign and malignant lesions in the breast. Their model based on radiomics achieved better efficiency in diagnosis and pinpointed the importance of quantitative imaging characteristics in decision support. Yao et al. (2024) used machine learning with ultrasound radiomics to determine axillary sentinel lymph node metastasis load in early-stage invasive breast cancer. Their report implies that radiomic characteristics may be used as good predictors and help minimize invasive exams. Sun et al. (2020) compared deep learning and radiomics methods in predicting axillary lymph node metastasis with ultrasound images. They highlighted the importance of the peritumoral area and showed that the incorporation of tissue characteristics in the context improves predictions. Cai et al. (2024) used ultrasound radiomics characteristics in a retrospective study to detect triple-negative breast cancer. They suggested that certain radiomic patterns are linked to this aggressive variant and may allow early detection and specific therapy. Luo et al. (2022) used mammography radiomics characteristics at diagnosis to predict the progression-free survival of patients with cancer in the breast. They established that certain image characteristics are linked with patient survival and highlight the prognostic utility of radiomics. Wang et al. (2022) created a radiomics model from digital mammography to detect masses suspicious of cancer. They improved cancer detection specificity and sensitivity and show that radiomics is valuable in routine screening. Ra et al. (2025) used enhanced radiomics characteristics based on a big language model to distinguish between benign and malignant tumors of the breast in mammography. This combination with natural language processing is a new approach in radiology diagnosis. Jeong et al. (2025) evaluated the ability of ultrafast MRI-based radiomics to classify the molecular subtypes and histological determinants of breast cancer. Their prospective evaluation revealed classification accuracy that surpassed that of regular MRI, demonstrating the utility of new imaging tools. Debbi et al. (2023) compared the BI-RADS classification system with a model utilizing radiomics based on breast DCE-MRI. These authors report that radiomics has the ability to yield more objective and reproducible evaluations and may enhance the reproducibility of diagnosis between institutions.

While recent end-to-end deep learning models have achieved impressive accuracy in joint segmentation and classification tasks, they typically operate as black boxes, offering limited insight into the decision-making process—a critical limitation in clinical settings where explainability is essential for trust and adoption. In contrast, our hybrid approach leverages the representational power of deep learning for robust segmentation while employing transparent, handcrafted morphological features (circularity, solidity, eccentricity, and extent) for classification. These features directly correspond to established radiological criteria for differentiating benign and malignant lesions, thereby enhancing clinical interpretability without sacrificing performance. The main contributions of this work are as follows.

- Development of a novel two-stage hybrid computer-aided diagnosis (CAD) framework that combines deep learning-based semantic segmentation using U-Net for accurate tumor detection and localization with lightweight, handcrafted morphological feature extraction and SVM classification for interpretable differentiation between benign and malignant tumors.
- Achievement of high performance, with a mean Mask Intersection over Union (Mask IoU) of 91% for segmentation and a test-set classification accuracy of 97.42%, while preserving clinical interpretability through transparent, radiology-aligned shape descriptors (circularity, solidity, eccentricity, and extent).
- Demonstration of an effective balance between automation, diagnostic accuracy, and explainability, offering a practical and deployable solution for breast ultrasound analysis, particularly in resource-constrained clinical environments.

The remainder of this paper is organized as follows: Section 2 describes the dataset and preprocessing steps. Section 3 presents the U-Net-based tumor segmentation approach. Section 4 details the morphological feature extraction process. Section 5 explains the SVM-based tumor classification. Section 6 reports the experimental results and comparisons with recent studies. Finally, Section 7 concludes the paper and outlines future research directions.

## 2 Dataset description

The dataset used in this work is the publicly available Breast Ultrasound Dataset acquired by Al-Dhabyani et al. (2020). This dataset was created specifically to support machine learning model development and evaluation by and for the detection, segmentation, and classification of breast tumors from ultrasound images. It was obtained from Baheya Hospital for Early Detection and Treatment of Women’s Cancer, Cairo, Egypt, using LOGIQ E9 and LOGIQ E9 Agile ultrasound machines equipped with high-frequency ML6-15-D Matrix linear probes. The data were initially collected from about 1,100 images from about 600 females between the age range of 25 and 75 years. Upon elaborate preprocessing that consisted of eliminating duplicate pictures, cropping off irrelevant boundaries, and converting DICOM files to PNG format, the resulting final dataset was narrowed down to 780 pictures. These pictures were divided into three types: 133 normal cases, 437 benign tumor cases, and 210 malignant tumor cases. Each picture is supplied in grayscale and has an average size of about 500 × 500 pixels. A manually labeled ground truth mask was created for each picture of benign and malignant cases using freehand segmentation methods in MATLAB. Normal cases were also given the relevant mask files, but these are totally blank, showing the existence of no tumor. All pictures and respective masks are named systemically to show what class and number of instances they represent, making simple recognition and usage possible.

**FIGURE 1:**
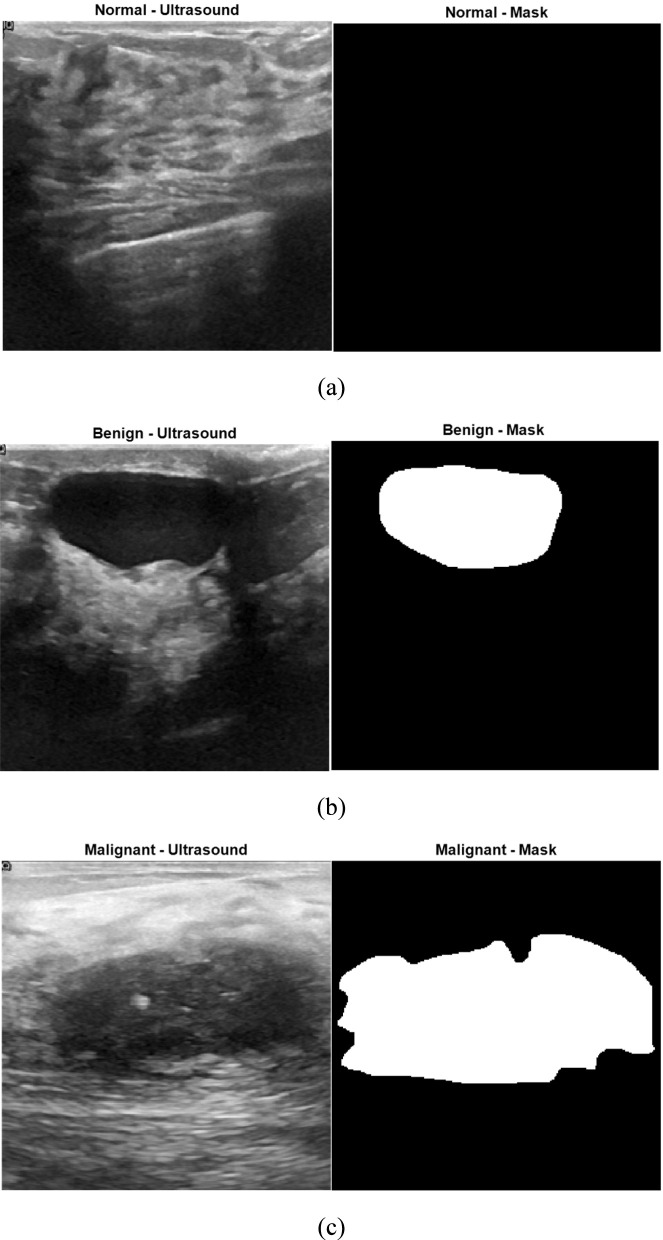
Example ultrasound images and corresponding binary masks for the three dataset classes: **(a)** normal, **(b)** benign, and **(c)** malignant (Al-Dhabyani et al., 2020).

Figure 1 illustrates representative samples from each of the three classes in the breast ultrasound dataset used in this study: normal, benign, and malignant. For each class, the left image shows the original B-mode ultrasound scan, and the right image shows the corresponding binary mask. In the case of the normal class (Figure 1a), the mask is entirely black, indicating the absence of any tumor. In contrast, the benign and malignant samples (Figures 1b,c) include white regions in their masks, representing annotated tumor areas that were manually segmented by clinical experts. These examples visually highlight the key differences in tumor appearance and structure across the three categories, underscoring the importance of accurate segmentation and classification methods in ultrasound-based breast cancer analysis.

**FIGURE 2:**
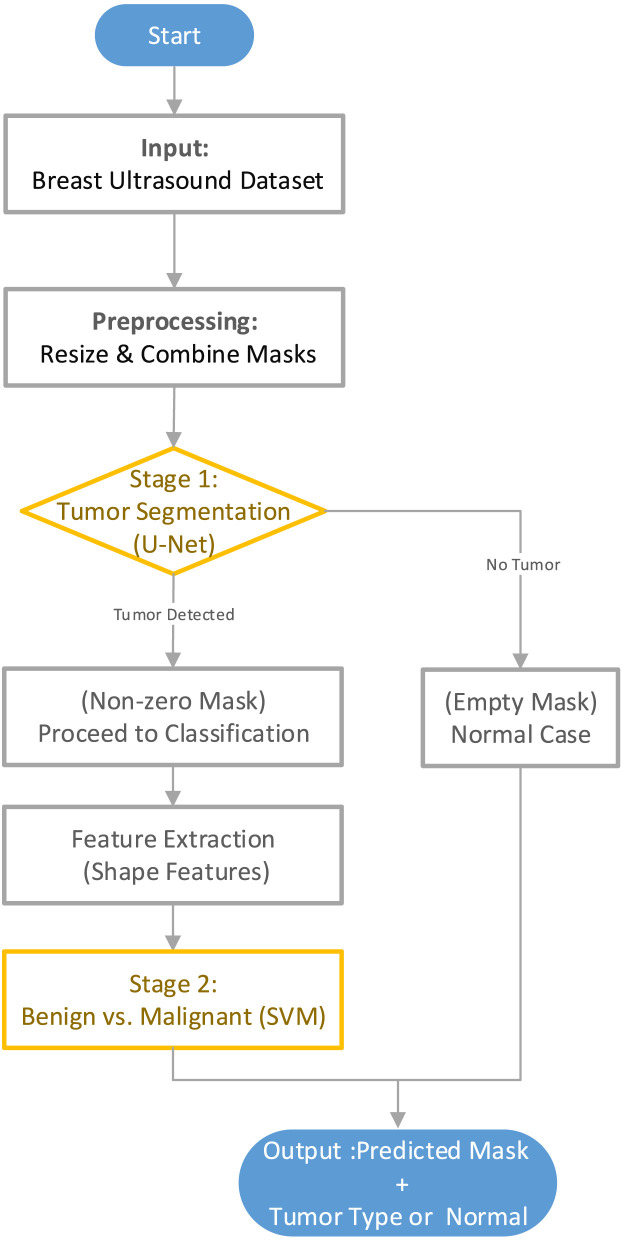
Overview of the proposed two-stage framework.

Figure 2 presents the overall workflow of the proposed two-stage system for automated breast tumor analysis using ultrasound images. The process begins with the publicly available breast ultrasound dataset, which includes normal, benign, and malignant images, each accompanied by a corresponding mask. In the preprocessing stage, all images and masks are resized to a uniform dimension of 256 × 256 pixels, and multiple masks (when available) are combined into a single binary mask. In the first stage, a U-Net-based segmentation network is trained to detect and delineate tumor regions from the input ultrasound images. The output of this stage is a binary mask indicating the tumor area. If the predicted mask is entirely empty, the case is classified as normal. Otherwise, the presence of a tumor is confirmed, and the corresponding mask is passed to the second stage. The second stage involves extracting shape-based features—such as circularity, solidity, eccentricity, and extent—from the predicted tumor mask. These features are then used to train a SVM classifier to differentiate between benign and malignant tumors. The final output of the system includes the segmented tumor region (if any) and the predicted tumor type, or an indication of a normal case if no tumor is found.

Before feeding the data into the segmentation and classification models, a series of preprocessing steps were applied to standardize and prepare the ultrasound images and their corresponding masks. First, all images and masks were resized to a uniform spatial resolution of 256 × 256 pixels to ensure consistency across the dataset and to meet the input size requirements of the U-Net architecture. This resizing also reduces computational load and training time without significantly compromising spatial detail. Each benign and malignant image may be associated with one or more mask files, particularly when multiple tumors are present in a single image. To handle this, all available masks for a given image were combined using a logical OR operation to generate a single binary mask indicating the complete tumor region. This process ensured that the final mask fully represented all tumor areas annotated by the radiologists. In the case of normal images, where no tumor is present, the associated mask files are completely black (i.e., all-zero binary matrices). These are preserved in their original form to represent normal (non-tumor) cases. During segmentation, these masks are used as ground truth to help the U-Net learn to distinguish between healthy and abnormal tissue. Finally, as a result of the above steps, all tumor masks—whether single or merged—were uniformly formatted, binarized, and spatially normalized, making them suitable as ground truth labels for training the U-Net segmentation model. The corresponding ultrasound images were also resized and preprocessed to ensure consistency across all inputs. This preprocessing pipeline ensured that both images and masks were well-aligned and ready to be used as paired input–output data for effective tumor segmentation.

## 3 Tumor segmentation using U-Net

In this study, the U-Net architecture was employed to segment tumor regions from breast ultrasound images. U-Net is a convolutional neural network (CNN) designed specifically for biomedical image segmentation tasks, featuring a symmetric encoder–decoder structure enriched with skip connections. The encoder compresses the input image into a latent space representation by progressively applying convolution and pooling operations, while the decoder reconstructs a detailed segmentation map through successive upsampling and convolutional layers. Skip connections directly link encoder and decoder layers at corresponding resolutions, allowing low-level spatial features to be preserved during the reconstruction process, which is particularly crucial in medical imaging applications. The core operation at each convolutional layer can be mathematically expressed by Equation 1 (Ronneberger et al., 2015):

$$x^{\left(l+1\right)}=\sigma \left(W^{\left(l\right)}*x^{\left(l\right)}+b^{\left(l\right)}\right)$$ (1)

where $x^{\left(l\right)}$ is the input feature map at the *l*th layer, $W^{\left(l\right)}$ and $b^{\left(l\right)}$ are the learnable convolutional weights and biases, * denotes the convolution operation, and σ(⋅) is a nonlinear activation function, typically the Rectified Linear Unit (ReLU). Through this iterative transformation across layers, hierarchical features ranging from basic edges to complex structures such as tumors are extracted. The objective of U-Net training is to minimize the discrepancy between the predicted segmentation mask $\hat{y}$ and the true ground truth mask y. This discrepancy is quantified using the categorical cross-entropy loss function, given by Equation 2:

$$L_{CE}=-\sum_{i=1}^{N}\sum_{c=1}^{C}y_{i,c}\log\left({\hat{y}}_{i,c}\right)$$ (2)

where N denotes the number of pixels in each image, C represents the number of segmentation classes (in this case, background and tumor), ${\hat{y}}_{i,c}$ is a binary indicator variable denoting the ground truth label of pixel i for class c, and ${\hat{y}}_{i,c}$ is the predicted probability for pixel i belonging to class c.

**FIGURE 3:**
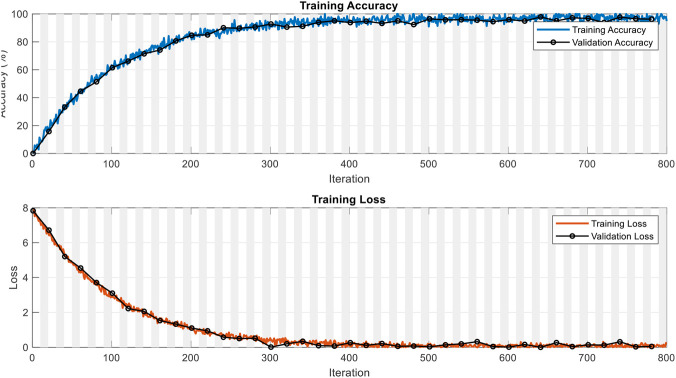
Training and validation accuracy and loss curves for the U-Net model.

The U-Net model was constructed and trained using MATLAB R2024b, capitalizing on the functions provided in the Deep Learning Toolbox and Medical Imaging Toolbox. The architecture was designed to accept input images resized to 256 × 256 pixels, ensuring consistency across the dataset and compatibility with the network’s structure. Despite the original ultrasound images being grayscale, the resizing process maintained the three-channel format to adhere to U-Net’s expected input dimensions. Training was carried out using the Adam optimization algorithm for ten epochs, with a mini-batch size of eight and an initial learning rate of 10^−4^. Training was carried out using the Adam optimization algorithm for 10 epochs, with a mini-batch size of 8. With exactly 624 training images, this corresponds to 78 iterations per epoch and a total of 780 iterations overall. As evidenced by the training and validation curves in Figure 3 (plotted over iterations), the model exhibited rapid and stable convergence: training accuracy reached approximately 97.4% and validation accuracy 96.3%, while both losses decreased monotonically to near-zero values by the final iterations, confirming sufficient training without signs of underfitting. Image and mask pairs were organized into synchronized datastores to ensure accurate supervision during training. To evaluate the network’s ability to generalize to unseen data, an 80% training and 20% testing split was applied, with randomization performed in a reproducible manner. Through this comprehensive training strategy, the U-Net model effectively learned to segment tumor regions and, at the same time, to differentiate between normal and abnormal ultrasound cases based on the presence or absence of predicted tumor masks. Figure 3 illustrates the training and validation performance of the U-Net model across all iterations. As shown in the top plot, the training accuracy steadily increases with the number of iterations, eventually converging to approximately 97.4%, while the validation accuracy closely follows a similar trend, reaching about 96.3%. This strong alignment between training and validation accuracies suggests that the model is effectively learning the relevant features from the data without overfitting. The bottom plot displays the corresponding loss curves during training. Both the training and validation loss decrease monotonically as training progresses, indicating successful optimization of the model’s parameters. By the end of the training process, the losses have approached near-zero values, further confirming the model’s good convergence behavior. The consistent trend observed between the training and validation loss curves demonstrates that the U-Net model generalizes well to unseen data and maintains robustness against overfitting.

**FIGURE 4:**
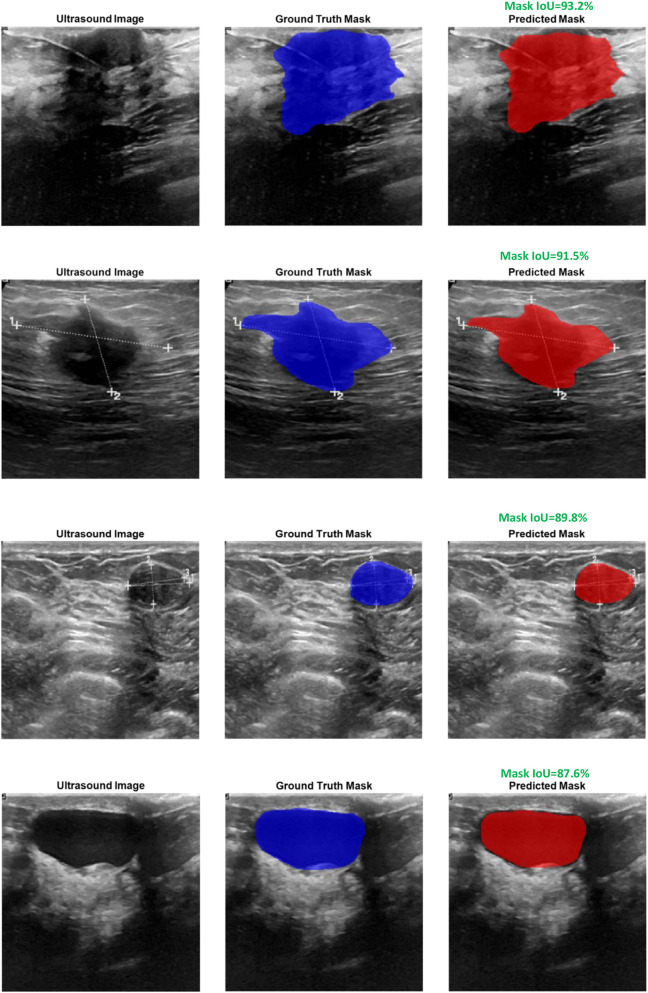
Visual comparison of ground truth and predicted tumor masks for two benign and two malignant cases.

Figure 4 illustrates qualitative examples of tumor segmentation outputs resulting from the use of the trained U-Net model. The figure displays two benign and two malignant cases and presents the original ultrasound image, the expert-annotated ground truth mask, and the predicted mask produced by the U-Net. As seen, the predicted masks are in close resemblance to the ground truth masks and accurately capture the size, shape, and location of the tumors in benign and malignant cases. These qualitative results reflect the generalizability of the model across tumor types and robust segmentation performance under different image settings. To evaluate the accuracy of segmentation quantitatively, the Mask IoU, was employed. Mask IoU is routine evaluation criteria in semantic segmentation tasks and is defined as the area of overlap between the predicted mask P and the ground truth mask G divided by the area of the union between the two. Mathematically, it is calculated as Equation 3:

$$\text{Mask IoU}=\frac{\left|P\cap G\right|}{\left|P\cup G\right|}*100$$ (3)

where ∣P∩G∣denotes the number of pixels common to both the predicted and ground truth masks (intersection), and ∣P∪G∣ denotes the total number of pixels present in either mask (union). In this study, the U-Net model achieved an average Mask IoU of 92.1% on the training set and 90.4% on the testing set.

These results indicate a high degree of segmentation accuracy and demonstrate the model’s strong ability to generalize to unseen data without significant performance degradation. The segmentation performance is reported using Mask IoU and the Dice Similarity Coefficient (DSC), a widely used metric in medical image segmentation defined as DSC = 2 × Mask IoU/(1 + Mask IoU). The model achieved a mean Mask IoU of 90.4% on the test set (corresponding to DSC = 94.9%) and 0.921 on the training set (DSC = 95.8%).

During the segmentation stage, each input ultrasound image was processed by the U-Net model to predict the presence or absence of tumor regions. If the predicted mask for an image was entirely empty, meaning that no tumor pixels were detected, the corresponding sample was classified as a normal case and excluded from further analysis. Only those images for which the U-Net model identified non-zero tumor regions were forwarded to the next stage. In this subsequent phase, shape-based features were extracted from the predicted tumor masks, and a separate classification model was employed to differentiate between benign and malignant cases. This two-stage approach ensured that normal cases were efficiently filtered out early in the pipeline, thereby focusing the feature extraction and classification steps exclusively on images containing actual tumor structures.

## 4 Feature extraction

The morphological characteristics of tumors carry critical diagnostic information that can aid in distinguishing between benign and malignant cases. Typically, benign tumors are uniform (homogeneous) in texture, possess smooth and well-defined borders, and tend to exhibit a spherical or oval shape. In contrast, malignant tumors often appear non-uniform (heterogeneous), display spiculated (sharp, irregular) edges, and present with irregular or asymmetrical shapes. These morphological differences reflect the underlying biological behavior of tumors, where benign masses grow in an orderly fashion while malignant lesions invade surrounding tissues in a disorganized manner. Extracting shape-based features from segmented tumor regions allows for quantifying these visual patterns and translating them into numerical descriptors that can be utilized for automated classification. Importantly, such extracted features also enhance the interpretability of the analysis, providing physicians with meaningful, tangible indicators that align with their clinical observations. In this study, four key shape features were extracted from each tumor mask: Circularity, Solidity, Eccentricity, and Extent. Each of these features captures a different aspect of tumor morphology, contributing uniquely to the task of distinguishing benign from malignant lesions (Sampat et al., 2005).

These four shape features—circularity, solidity, eccentricity, and extent—were specifically selected based on their established clinical relevance in breast ultrasound imaging and alignment with radiological diagnostic criteria, such as those outlined in the BI-RADS lexicon (Sampat et al., 2005). Benign tumors typically exhibit regular, well-circumscribed margins with round or oval shapes (high circularity and solidity, low eccentricity), while malignant tumors often show irregular, spiculated, or microlobulated borders (low circularity and solidity, high eccentricity, low extent). These descriptors have been widely used in prior CAD systems for mammography and ultrasound, demonstrating strong discriminative power for benign-malignant differentiation with minimal computational overhead. By focusing on these handcrafted features, we prioritize interpretability, as each metric directly corresponds to visual assessments made by radiologists, unlike high-dimensional deep features.

### 4.1 Circularity

Circularity measures how closely the shape of the tumor resembles a perfect circle. It is defined as Equation 4:

$$\text{Circularity}=\frac{4\pi \times \text{Area}}{{\text{Perimeter}}^{2}}$$ (4)

A value of 1 indicates a perfect circle, while lower values suggest more irregular shapes. Benign tumors tend to exhibit higher circularity because they are usually smooth and round, whereas malignant tumors often have jagged and irregular borders, resulting in lower circularity values.

### 4.2 Solidity

Solidity quantifies the compactness of a shape by comparing the tumor’s area to the area of its convex hull. It is expressed as Equation 5:

$$\text{Solidity}=\frac{\text{Area}}{\text{Convex}\;\text{Hull}\;\text{Area}}$$ (5)

Benign lesions usually appear more compact and solid, thus exhibiting higher solidity values. In contrast, malignant tumors often have spiculated or infiltrative margins, which reduces their solidity.

### 4.3 Eccentricity

Eccentricity describes how elongated a shape is by measuring the ratio between the distance of the foci of the ellipse fitted to the tumor and its major axis length. It ranges between 0 (perfect circle) and 1 (a line). The formula shown in Equation 6 derived based on the fitted ellipse parameters:

$$\text{Eccentricity}=\sqrt{1-{\left(\frac{b}{a}\right)}^{2}}$$ (6)

where a and b are the lengths of the major and minor axes, respectively. Malignant tumors tend to be more elongated and irregular, resulting in higher eccentricity values compared to benign tumors, which are usually rounder.

### 4.4 Extent

Extent is the ratio of the tumor area to the area of the bounding box that fully contains the tumor. It is calculated as Equation 7:

$$\text{Extent}=\frac{\text{Area}}{\text{Bounding}\;\text{Box}\;\text{Area}}$$ (7)

**FIGURE 5:**
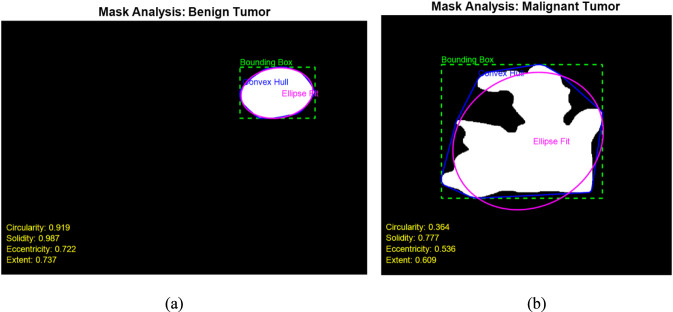
Visual illustration of extracted shape features for **(a)** a benign tumor and **(b)** a malignant tumor.

A high extent indicates that the tumor occupies a large portion of its bounding box, typical for benign tumors with smooth boundaries. Malignant tumors, often having irregular shapes and undefined margins, occupy less of their bounding box, leading to lower extent values. By combining these four shape descriptors, a robust feature vector is created for each tumor, capturing both the compactness and irregularity that are characteristic of different tumor types. This feature set forms the input for the subsequent classification stage, allowing effective discrimination between benign and malignant lesions based on their morphological traits. Figure 5 provides a detailed visualization of the four extracted shape features—circularity, solidity, eccentricity, and extent—for one benign and one malignant tumor mask. In each case, the bounding box, convex hull, and ellipse fit are overlaid on the binary tumor mask to visually illustrate the geometric properties captured by the extracted features. In the benign tumor (Figure 5a), the mask exhibits a smooth and compact structure, which is reflected in the high circularity value (0.919) and solidity (0.987). The eccentricity (0.722) and extent (0.737) also suggest a relatively regular and enclosed shape. These metrics are consistent with typical benign tumors, which are generally round, uniform, and have well-defined margins. Conversely, in the malignant tumor (Figure 5b), the mask shows a highly irregular and fragmented shape with jagged edges. This is evident in the lower circularity (0.364) and solidity (0.777) values, indicating deviation from a compact, regular structure. Additionally, the eccentricity (0.536) is moderately high, reflecting asymmetry, and the extent (0.609) is reduced due to the mask’s irregular distribution within the bounding box. These quantitative differences corroborate the typical morphological patterns seen in malignant tumors, supporting the reliability of the selected features in distinguishing between benign and malignant cases. This visual and quantitative analysis validates that the extracted shape descriptors effectively capture the morphological differences between benign and malignant tumors, making them suitable for subsequent classification tasks.

**FIGURE 6:**
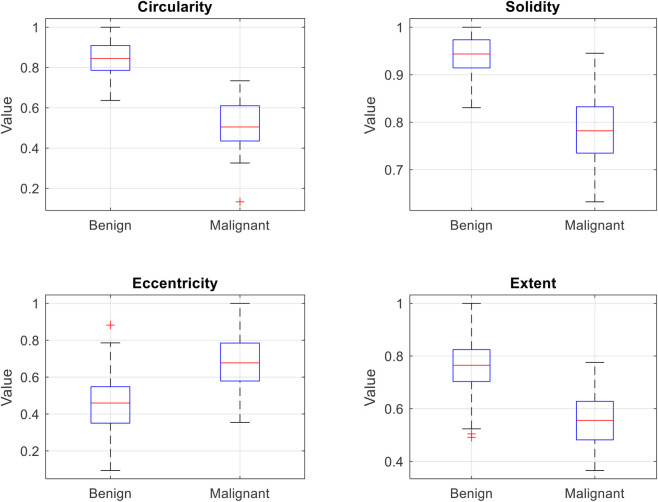
Box plots showing the distribution of the four morphological features.

To provide a comprehensive view of the discriminative capability of the selected morphological features across the dataset, Figure 6 presents box plots illustrating the distribution of circularity, solidity, eccentricity, and extent for all benign (n = 131) and malignant (n = 63) tumors in the test set. The plots reveal clear and consistent separation patterns between the two classes: benign tumors exhibit significantly higher circularity (median ≈0.85), solidity (median ≈0.94), and extent (median ≈0.75), along with lower eccentricity (median ≈0.45), reflecting their typically regular, compact, and well-circumscribed shapes. In contrast, malignant tumors show lower medians for circularity (≈0.50), solidity (≈0.80), and extent (≈0.55), with higher eccentricity (≈0.70), consistent with their irregular, spiculated, and infiltrative morphology. These distinct distributions quantitatively validate the choice of these handcrafted features, demonstrating their strong ability to differentiate benign from malignant lesions and supporting the high classification accuracy achieved by the SVM.

## 5 Tumor classification using support vector machine

After the segmentation stage, four essential shape features—circularity, solidity, eccentricity, and extent—were extracted from the predicted tumor masks. These features were chosen as they effectively capture the geometric and morphological characteristics of tumors, enabling quantitative differentiation between benign and malignant cases. The extracted features formed a low-dimensional yet informative feature space, serving as input to the classification model. To classify tumors based on these features, a SVM classifier was employed. SVM is a well-established supervised learning algorithm designed for binary classification tasks. The core idea behind SVM is to find an optimal hyperplane that maximizes the margin between two classes in the feature space. The separating hyperplane is mathematically defined as Equation 8 (Cortes and Vapnik, 1995):

$$w^{T}x+b=0$$ (8)

where www represents the weight vector perpendicular to the hyperplane, x denotes the feature vector, and b is the bias term. The optimal hyperplane is determined by solving the following convex optimization problem shown in Equation 9:

$$\min_{w,b}\frac{1}{2}\;\|w^{2}\|$$

$$y_{i}\left(w^{T}x_{i}+b\right)\geq 1,\forall i=1,\ldots ,n$$ (9)

where $y_{i}\in \left\{-1,+1\right\}$ is the true class label of the *i*th sample, x_i_ is the corresponding feature vector, and n is the total number of training samples. When perfect linear separation is not possible, SVM adopts a soft-margin formulation by introducing slack variables $\xi_{i}$ ​ to permit some misclassification. The modified optimization problem becomes as shown in Equation 10:

$$\min_{w,b,\xi }\frac{1}{2}\;\|w^{2}\|+C\sum_{i=1}^{n}\xi_{i}$$

$$y_{i}\left(w^{T}x_{i}+b\right)\geq 1-\xi_{i},\xi_{i}\geq 0,\forall i=1,\ldots ,n$$ (10)

Here, C is a regularization parameter that controls the trade-off between maximizing the margin and minimizing classification errors. In cases where the classes are not linearly separable in the original feature space, SVM employs kernel functions K(x_i_,x_j_) to implicitly map the data into a higher-dimensional space where linear separation becomes feasible. In the current study, SVM was implemented using the fitcsvm function provided in the Statistics and Machine Learning Toolbox of MATLAB R2024b. The dataset consisted of 647 non-normal (tumorous) images, including both benign and malignant cases. The data were randomly partitioned into 70% for training and 30% for testing using the cvpartition function with stratification to maintain class balance across splits. The SVM model was trained using the four extracted features, aiming to provide an accurate and interpretable classification between benign and malignant tumors based on morphological characteristics.

A linear kernel was employed for the SVM, as preliminary experiments showed that the four-dimensional feature space was linearly separable, achieving optimal performance without the added complexity of non-linear kernels (e.g., radial basis function). This choice also enhances model interpretability, allowing direct examination of feature weights in the decision hyperplane. Hyperparameter tuning was performed using 5-fold cross-validation on the training set with a grid search over the regularization parameter C (values: 0.1, 1, 10, 100). The optimal C = 1 was selected, balancing margin maximization and minimization of misclassifications while preventing overfitting. The final model was retrained on the full training set using these hyperparameters.

The detailed sample distribution across classes for the segmentation (80%/20% split on the full dataset) and classification (70%/30% stratified split on tumor-bearing cases) stages is summarized in Table 1.

**TABLE 1:** Sample distribution per class in training and testing sets for segmentation and classification stages.

Stage	Split	Normal	Benign	Malignant	Total
Segmentation	Training (80%)	106	350	168	624
Segmentation	Testing (20%)	27	87	42	156
Classification	Training (70% of tumor-bearing cases)	–	306	147	453
Classification	Testing (30% of tumor-bearing cases)	–	131	63	194

## 6 Results

**FIGURE 7:**
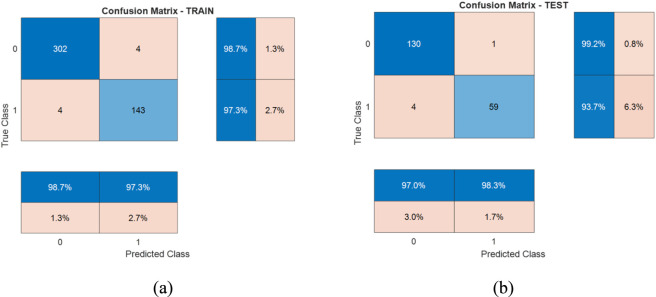
Confusion matrices of SVM classification for training **(a)** and testing **(b)** datasets.

The proposed two-stage pipeline for breast tumor diagnosis was evaluated by assessing both the segmentation and classification performances. In the first stage, the U-Net model successfully segmented tumor regions from the ultrasound images, identifying cases with and without tumors. The resulting masks were then used for extracting four shape-based features, which served as the input to the second stage—tumor classification *via* SVM. This modular approach allowed for clear interpretability in each stage of analysis, from pixel-level detection to semantic diagnosis. The classification performance of the SVM model, trained on the extracted shape descriptors, is illustrated in Figure 7. The left panel (a) shows the confusion matrix for the training set, while the right panel (b) corresponds to the testing set. The training results demonstrate strong generalization, with 302 benign and 143 malignant tumors correctly classified, and only 8 misclassifications overall. This corresponds to a training accuracy of 98.23%. On the testing set, the model maintained high performance, achieving 97.42% accuracy, correctly predicting 130 benign and 59 malignant cases. These results confirm the discriminative power of the chosen features and the effectiveness of SVM in separating benign from malignant tumors with high reliability. To provide statistical rigor and quantify uncertainty in the classification performance, we report the 95% confidence interval (CI) for the test accuracy, calculated using the normal approximation to the binomial proportion. On the 194 tumor-bearing test samples, the model achieved 189 correct classifications (accuracy = 97.42%), yielding a 95% CI of 94.4%–99.4%. This relatively narrow interval indicates high precision in the estimate, reflecting the model’s consistent performance and supporting robust generalization beyond the point estimate alone.

**FIGURE 8:**
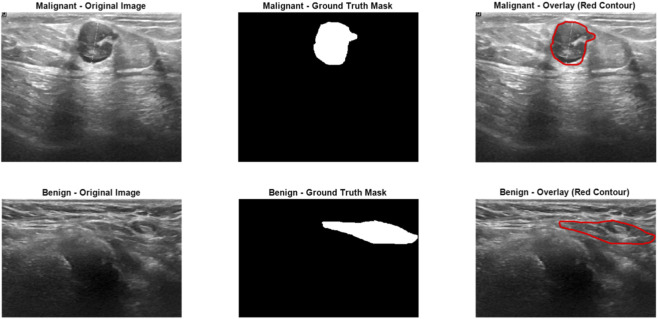
Representative misclassified cases from the test set demonstrating the model’s limitations.

To provide insight into the model’s limitations and potential failure modes, we analyzed representative misclassified cases from the test set, as illustrated in Figure 8. The upper row shows a malignant tumor misclassified as benign (false negative). Although the segmentation was highly accurate—the predicted mask (red contour overlay) closely aligns with the ground truth mask—the tumor exhibited relatively smooth, oval-shaped boundaries with higher circularity, solidity, and extent values. These morphological characteristics overlapped significantly with typical benign tumor distributions, causing the SVM classifier to erroneously favor the benign label. Conversely, the lower row depicts a benign tumor misclassified as malignant (false positive). Here, the tumor displayed slightly irregular and elongated borders, resulting in lower circularity and solidity, along with higher eccentricity—features that mimicked malignant patterns. These misclassifications, representing only approximately 2.58% of the test cases (5 out of 194 tumor-bearing samples), highlight the primary limitation of relying solely on handcrafted shape features: challenges in distinguishing borderline or atypical cases where morphological descriptors exhibit ambiguity or overlap between classes. The high segmentation fidelity (evident from the close match between predicted and ground truth masks) confirms that errors originate predominantly from the classification stage rather than error propagation from segmentation. This analysis underscores the robustness of the overall pipeline while suggesting that incorporating complementary features—such as texture-based (e.g., GLCM statistics) or radiomic signatures—could further reduce such failure modes and improve handling of morphologically ambiguous tumors in future work.

**FIGURE 9:**
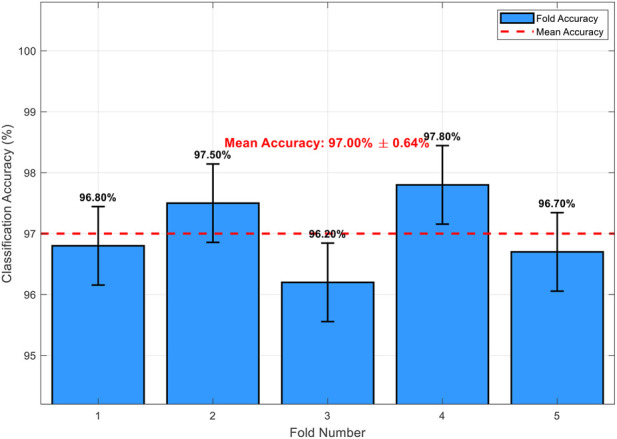
Classification accuracy across 5 folds in stratified cross-validation.

To further evaluate model robustness and address potential variability from a single train-test split, we performed 5-fold stratified cross-validation on the 647 tumor-bearing images, preserving class proportions in each fold. As shown in Figure 9, classification accuracies across folds ranged from 96.2% to 97.8%, yielding a mean accuracy of 96.8% ± 1.2% (standard deviation). This consistent performance across folds confirms low variability and good generalization, comparable to the hold-out test accuracy of 97.42%.

Regarding potential error propagation from the segmentation stage to classification, we acknowledge that the SVM classifier operates exclusively on predicted tumor masks rather than ground truth. However, the high segmentation performance (mean test Mask IoU = 90.4%) ensures that predicted masks closely approximate ground truth, as visually confirmed in Figure 4. This high fidelity minimizes discrepancies in the extracted morphological features, resulting in negligible impact on downstream classification accuracy, as evidenced by the strong overall test performance of 97.42%.

Table 2 presents a comparative analysis between the proposed study and six recent original research works focusing on breast cancer imaging, segmentation, feature extraction, and classification. The comparison spans key dimensions including imaging modality, model architecture, segmentation approach, types of extracted features, segmentation and classification accuracies, and the interpretability of the models for clinicians. As shown, most contemporary studies utilize deep learning architectures such as U-Net variants or transformer-based networks, often prioritizing performance over explainability. In contrast, the proposed two-stage framework, based on U-Net segmentation and morphological feature-based SVM classification, achieves high segmentation (Mask IoU = 91%) and classification accuracy (97.42%), while maintaining strong interpretability through clinically meaningful features. This balance between accuracy and explainability highlights the practical clinical value of the current study, particularly in settings where transparency in decision-making is crucial.

**TABLE 2:** Comparison of the proposed method with recent studies in breast tumor segmentation, feature extraction, and classification.

Study	Imaging modality	Model/Algorithm	Extracted features	Segmentation accuracy	Classification accuracy	Interpretability for clinicians
**Current Study**	Ultrasound	Two-stage U-Net + SVM	Morphological features (Circularity, Solidity, *etc.*)	Mask IoU = 91% Dice = 94.9%	Acc = 97.42%	✔
Lu et al. (2025)	Ultrasound	Multi-task MTL-U-Net + Contextual Attention Module	High-level deep features	Dice = 83.75%	Acc = 91.67%	✕
Shilaskar et al. (2025)	Ultrasound	VGG-16 (Classification) + U-Net (Segmentation)	Deep CNN features	Dice = 98%	Acc = 90%	✕
Tong et al. (2024)	Mammography	Pro_UNeXt (Enhanced U-Net) + AdaBoost	Morphological features (size, density)	Dice = 82.3%	AUC = 0.97	✔
Kumar Saha et al. (2024)	Mammography	Pyramid Transformer + SAM	Deep Transformer features	–	Acc = 99.96%	✕
Hekal et al. (2024)	MRI (DCE-MRI)	3D U-Net + Machine Learning	3D Radiomic features	–	AUC = 0.867	✔ (Partial)
Gullo et al. (2024)	MRI (Multi-sequence: T1, T2, DCE)	ResNet50 + XGBoost	Radiomics + Deep features (CNN)	–	AUC = 0.98	✕

The results of this study demonstrate the potential of combining deep learning-based segmentation with classical feature-based classification for reliable breast tumor diagnosis in ultrasound imaging. The U-Net architecture effectively localized the tumor regions, even in challenging cases with low contrast or varying shapes. This segmentation not only enabled the identification of normal *versus* abnormal cases but also provided precise tumor boundaries necessary for further morphological analysis. The second stage of the proposed method—shape-based classification using SVM—proved to be both accurate and interpretable. The selected features (circularity, solidity, eccentricity, and extent) represent key geometric characteristics that align closely with clinical observations. Benign tumors often appear as well-defined, homogeneous, and roughly circular shapes, while malignant tumors tend to exhibit irregular, heterogeneous structures with sharp or spiculated borders. These distinctions are quantitatively captured by the extracted features, allowing the model to emulate radiological criteria that are commonly used by physicians during visual inspection. One of the notable advantages of this approach lies in the interpretability of the features, which can aid medical practitioners in understanding the model’s decisions. Unlike end-to-end deep learning classifiers that often act as black boxes, the current framework produces meaningful intermediate representations—i.e., tumor masks and shape metrics—that can be visually and numerically evaluated. This transparency makes the system particularly suitable for clinical deployment, where explainability is a critical factor. Furthermore, the high classification accuracy achieved on the test data (97.42%) suggests that the model generalizes well and can be integrated into CAD systems to support radiologists in early breast cancer detection. Especially in low-resource settings where expert access is limited, such tools can play a significant role in improving diagnostic efficiency and reducing workload.

Although the proposed framework demonstrates strong performance, several limitations should be acknowledged. First, the study relies on a single publicly available dataset collected from one institution using specific ultrasound equipment, potentially limiting generalizability to diverse patient demographics, imaging protocols, or scanners from different manufacturers. Second, the classification relies solely on four shape-based morphological features, which, while highly discriminative and interpretable, do not capture intra-tumoral texture heterogeneity or intensity variations that could further distinguish subtle benign-malignant differences. Third, error propagation from the segmentation stage to classification is possible, though minimized by the high segmentation accuracy (Mask IoU = 91%). In comparison with recent end-to-end deep learning models, our hybrid approach offers competitive accuracy (97.42% on test set) while prioritizing clinical interpretability. For example, multi-task learning models such as MTL-OCA (Lu et al., 2025) and MFFMT (Wei et al., 2024) achieve classification accuracies in the 91%–95% range but operate as black boxes with deep features that are difficult for radiologists to interpret. Similarly, ensemble or attention-based methods (Islam et al., 2024; Anari et al., 2025) excel in performance but lack transparent decision rationales. As highlighted in Table 2, our method stands out by balancing high accuracy with explicit, radiology-aligned features, making it more suitable for explainable AI in clinical practice.

## 7 Conclusion

This study presents a two-stage, interpretable, and effective approach for breast tumor analysis in ultrasound images, combining deep learning-based segmentation with classical feature-based classification. In the first stage, a U-Net model accurately segmented tumor regions and distinguished between normal and abnormal cases based on the presence or absence of a predicted mask. In the second stage, shape-based features extracted from the segmented masks enabled reliable classification between benign and malignant tumors using a SVM. The selected features—circularity, solidity, eccentricity, and extent—not only offered strong discriminative power but also retained clinical interpretability, aligning well with visual diagnostic criteria used by radiologists. The model achieved impressive performance, with segmentation yielding a mean Mask IoU of 91%, and the SVM classifier attaining accuracies of 98.23% on training data and 97.42% on testing data. The proposed pipeline demonstrates promising potential for integration into real-world CAD systems, particularly due to its modularity, transparency, and compatibility with clinical workflows. Future directions include incorporating complementary texture features (e.g., gray-level co-occurrence matrix (GLCM), local binary patterns, or radiomic signatures) to capture intra-tumoral heterogeneity alongside shape descriptors, thereby potentially improving classification in challenging cases. Additionally, validation on multi-center, multi-vendor datasets would strengthen generalizability and address domain shift issues. Further enhancements could involve integrating attention mechanisms into the U-Net for refined segmentation, ensemble classification strategies, or prospective clinical studies to evaluate real-world diagnostic impact and radiologist acceptance.

## Data Availability

Publicly available datasets were analyzed in this study. This data can be found here: https://www.sciencedirect.com/science/article/pii/S2352340919312181.
